# 94. Psoriatic arthritis, rheumatoid arthritis or both?

**DOI:** 10.1093/rap/rky032.002

**Published:** 2018-09-20

**Authors:** Jean Zhang, Richard Smith, Sarah Bartram, Zoe Cole, Aisling Coy

**Affiliations:** Rheumatology, Salisbury Foundation Trust, Salisbury, UNITED KINGDOM

**Introduction:** Psoriatic arthropathy (PsA) is a common diagnosis particularly in those with known psoriasis presenting with inflammatory arthropathy. The main stay of initial management for PsA is similar in nature to that of rheumatoid arthritis (RA) starting with disease modifying agents such as methotrexate, sulphalazaine or leflunomide. Traditionally the first line biologic therapy for PsA and RA were anti-TNF inhibitors. For several years RA has had a number of alternative mode of action biologic therapies such as tocilizumab, rituximab, and abatacept, two of which may now be used first line. It is only recently that new biologics have emerged with alternative modes of action for psoriasis and PsA. These include secukinumab (anti-IL17) and ustekinumab (anti-IL12/23). These new biologics have been shown to treat both the skin and joints in PsA. Neither of these biologics are licensed for the treatment of RA. The newest generation of advanced treatment for inflammatory arthritis are the small molecules. The JAK inhibitor, tofacitinib is licensed for RA and has shown efficacy in clinical trials for PsA. We present a case of a middle aged gentleman with lifelong, severe psoriasis, referred by dermatology with an oligoarthritis. He was diagnosed and treated as PsA with initial treatment for PsA being moderately effective including TNF alpha inhibition. Due to skin deterioration he was switched to a PsA specific biologic therapy with resolution of skin but severe deterioration of joints, culminating in development of a rapidly progressive symmetrical polyarthopathy with development of nodules at his elbows side by side with psoriatic plaques.

**Case description:** A 57 year old male with lifelong severe psoriasis was referred in 2005 by the dermatologists to rheumatology because of the onset of arthralgia. His psoriasis was extensive with hyperkeratotic plaques involving knees, elbow, buttock, natal cleft, scrotum and groin. In addition he had dystrophic nails. His previous dermatology treatment included PUVA and a variety of topical ointments. He was found to have four tender and swollen joints and was commenced on methotrexate with moderate improvement. In 2006, the methotrexate was titrated up to 20mgs once weekly before being converted to subcutaneous methotrexate because of side effects. At this stage he had 33 tender joints and 13 swollen joints. His joints settled and his skin showed moderate improvement. In 2008, his methotrexate was increased to 25mgs subcutaneous because of a flare in his psoriasis. In 2014 he was commenced on golimumab in combination with methotrexate, chosen because the patient was not keen on injections. His joints responded well but his psoriasis did not fully respond. In 2015, his biologic therapy was switched to adalimumab and he was referred to the biologics clinic. On assessment he has a PASI score of 7.9 and a swollen joint count of 2 and a tender joint count of 1. His main concern was his skin and it was jointly agreed to change treatment to re-focus on his skin. He was switched to ustekinumab. Six months after initiation of ustekinumab his PASI was 1.6 but his arthritis had significantly deteriorated, he had a swollen and tender joint count of 20. He responded to an IM corticosteroid injection but one month later his joint count remained the same and he had developed plantar fasciitis. He was commenced on daily oral prednisolone and consideration was made to increase his ustekinumab to 90mgs every 3/12 but his weight fell below 100kg. His skin remained clear. He was subsequently switched to apremilast and then secukinumab, neither of which he was able to tolerate. During this time he developed a markedly elevated CRP and was investigated for possible lymphoma. This was excluded and the raised CRP was attributed to his active arthritis. In his most recent biologics assessment his joints were assessed both clinically and on ultrasound. The symmetrical nature and the ultrasound appearance (florid synovitis of MCPJs and wrists with possible erosions) was deemed more consistent with RA than PsA. Further examination revealed nodules over both elbows, next to his psoriatic patches, which according to the patient had developed over the last three months. Radiographs now showed erosions and his ACPA and RF were positive. He was commenced on tofactinib with (to date) good response.

**Discussion:** The patient presented with a history of severe, resistant to treat psoriasis and the onset of arthritis. A rheumatoid latex test was performed which was positive but the diagnosis was deemed to be PsA presumably due to the severe cutaneous disease. PsA is a clinically heterogeneous disorder. Five subtypes of PsA were recognised by Moll and Wright: oligoarthritis, polyarthritis, distal interphalangeal (DIP) joint predominant disease, psoriatic spondylitis and/or sacroiliitis, and arthritis mutilans. The CASPAR criteria for diagnosis of psoriatic arthritis has a negative test for rheumatoid (rheumatoid factor) as a diagnostic criteria and indeed a positive rheumatoid factor or ACPA may be helpful in determining rheumatoid arthritis with co-existing psoriasis from polyarticular psoriatic arthritis. However In a study by Perez-Alamino et al, Anti-CCP antibodies were found in 13.5% of a cohort of patients with Psoriatic arthritis and in a further paper by Punzi et al state that RF was found in 5% to 13% of patients with PsA. In our patient, the focus of management was initially biased towards treatment of his most debilitating condition (cutaneous psoriasis) whilst also treating his arthritis. This was not an issue initially because of the cross over in treatment modalities, however when he developed a secondary inadequate response to his TNF alpha inhibitor (psoriasis flare) the use of a psoriatic specific biologic led to the onset of a more polyarticular symmetrical form of erosive arthritis and an elevated CRP. In addition the patient developed nodules on his elbows consisted with RA, next to his established psoriasis! It is frequently taught in medical school to examine the elbows of patients with polyarthritis to look for psoriasis to make the diagnosis of PsA and differentiate form RA. It is rare to see both RA nodules and psoriatic plaques. The failure to respond to PSA specific biologic therapy led to the re-diagnosis of RA in out patient. Fortunately, the development of new agents such as the small molecule tofacitinib may treat both inflammatory forms of arthritis as well as cutaneous psoriasis.

**Key Learning Points**: Initial treatment for both PsA and RA is similar including traditional first line biologic therapy in the form of TNF alpha inhibition. Newer second line therapy using alternative modes of action may help differentiate what is PsA with a positive rheumatoid factor or RA with co-existing psoriasis. In our patient ultrasound was useful in differentiating RA from PsA. In our patient the development of nodules and positive serology confirmed the diagnosis of RA Modern therapies in the form of the Jak kinase inhibitor tofacitinib have shown efficacy in PsA, psoriasis and RA.

**Disclosure: J. Zhang:** None. **R. Smith:** Honoraria; Talks for Abbvie, BMS, MSD, Pfizer). Grants/research support; PI for Pharma sponsored research (BMS, Abbvie, Roche). **S. Bartram:** None. **Z. Cole:** None. **A. Coy:** None.

